# Repeatability of Semi-Quantitative and Volumetric Features from Artificial-Intelligence-Guided Lesion Segmentation on ^18^F-DCFPyL PSMA-PET/CT Images: Results from a Test-Retest Cohort

**DOI:** 10.3390/tomography12030038

**Published:** 2026-03-11

**Authors:** Md Zobaer Islam, Timothy G. Perk, Amy Weisman, Mark C. Markowski, Kenneth J. Pienta, Young E. Whang, Matthew I. Milowsky, Martin G. Pomper, Nicholas Wisniewski, Ralph A. Bundschuh, Rudolf A. Werner, Michael A. Gorin, Steven P. Rowe

**Affiliations:** 1 Department of Radiology, University of Texas Southwestern Medical Center, Dallas, TX 75390, USA; 2 AIQ Solutions, Madison, WI 53717, USA; 3 Sidney Kimmel Comprehensive Cancer Center, Johns Hopkins University School of Medicine, Baltimore, MD 21287, USA; 4 Division of Oncology, Department of Medicine, University of North Carolina, Chapel Hill, NC 27599, USA; 5 Department of Radiology, University of North Carolina, Chapel Hill, NC 27599, USA; 6 Department of Nuclear Medicine, Universitätsklinikum Carl Gustav Carus at the TU Dresden, 01307 Dresden, Germany; 7 German Cancer Consortium (DKTK), Partner Site Dresden, 01307 Dresden, Germany; 8 Department of Nuclear Medicine, LMU University Hospital, LMU Munich, 80539 Munich, Germany; 9 Milton and Carroll Petrie Department of Urology, Icahn School of Medicine at Mount Sinai, New York, NY 10029, USA

**Keywords:** PSMA-PET/CT, test–retest, PET features, artificial intelligence

## Abstract

**Objectives:** This study evaluated the test–retest repeatability of semi-quantitative and volumetric features derived from artificial intelligence (AI)-assisted lesion segmentation on ^18^F-DCFPyL Prostate Specific Membrane Antigen (PSMA)-PET/CT imaging of patients with prostate cancer (PCa). Specifically, we assessed the reliability of maximum, minimum and total standardized uptake values (SUV_max_, SUV_mean_, SUV_total_) and lesion volume measurements across varying lesion sizes and explored the implications of variability for clinical decision-making. **Methods:** We analyzed ^18^F-DCFPyL PSMA-PET/CT images from 22 patients with metastatic PCa. Lesion segmentation was performed using the AI-guided TRAQinform IQ technology, followed by a manual review to eliminate potential false-positive sites of uptake. Lesion-level test–retest repeatability was evaluated using 95% limits of agreement (LOA), intra-class correlation coefficient (ICC), within-subject coefficient of variation (wCOV) and Bland–Altman analysis for SUV and volumetric parameters. Lesions were stratified by size (>1 cm^3^ and >1.5 cm^3^) to assess the impact of lesion volume cut-offs on measurement variability. **Results:** A total of 297 lesions were analyzed, including 191 lesions > 1 cm^3^ and 161 lesions > 1.5 cm^3^. Test–retest variability was higher in smaller lesions, with narrower LOA and lower wCOV for larger lesions. SUV_max_ and SUV_mean_ exhibited lower variability than SUV_total_ and lesion volume. The 95% LOA for SUV_max_ ranged from −33.81% to +38.02% for all lesions, improving to −31.82% to +31.01% for lesions > 1.5 cm^3^. Similar trends were observed for SUV_mean_, SUV_total_, and volume. Bland–Altman plots confirmed reduced variability in larger lesions, with no significant systematic bias. **Conclusions:** The test–retest repeatability of AI-assisted PSMA-PET/CT features varies by feature type, with semi-quantitative features demonstrating improved repeatability relative to volumetric features. Additionally, repeatability is influenced by lesion size, with larger lesions exhibiting greater reliability. These findings highlight the importance of lesion size-dependent thresholds in response assessment and variability-aware feature selection in prognostic models. Current algorithms may be better optimized for larger lesions and higher volumes of disease, with limitations remaining in the robust detection and segmentation of smaller/more subtle lesions.

## Simple Summary

To date, the test–retest repeatability of lesion-level features derived from artificial intelligence (AI)-guided prostate-specific membrane antigen (PSMA)-PET lesion segmentation has not been systematically assessed. One reason for that is the lack of available data. We demonstrate that a unique test–retest dataset of PSMA-PET scans, i.e., paired scans of patients with metastatic prostate cancer obtained within one week of each other, provides a test bed for AI algorithms to demonstrate how repeatably they can identify and delineate tumors. The methodology we describe is a new means of assessing the validity of AI algorithms.

## 1. Introduction

Prostate cancer (PCa) is one of the most commonly diagnosed malignancies among men worldwide and remains a major cause of cancer-related morbidity and mortality, particularly in advanced and metastatic stages [1,2]. Accurate detection, staging, and longitudinal assessment of disease burden are essential for guiding treatment selection, evaluating therapeutic response, and improving patient outcomes [3]. Prostate-specific membrane antigen (PSMA)-targeted positron emission tomography/computed tomography (PET/CT) imaging has emerged as a highly sensitive and specific modality for detecting and characterizing PCa, particularly in metastatic and recurrent disease settings [4,5,6]. By leveraging the high expression of PSMA in PCa cells, PSMA-PET/CT enables precise localization of tumor lesions, aiding in disease staging, treatment planning, radiation dose estimation, and therapeutic response assessment [7,8,9]. Despite these advantages, the reliability and reproducibility of quantitative and volumetric metrics derived from PSMA-PET/CT images remain critical but uncertain factors in ensuring accurate disease monitoring and therapy evaluation [10]. Understanding the test–retest repeatability of those features is essential to establishing their robustness for clinical and research applications.

One of the major challenges in PSMA-PET/CT analysis is the limited availability of histologic ground truth for metastatic tumor volume, making it difficult to biologically validate segmentation results. Traditionally, semi-automated or manual delineation of tumor lesions by expert radiologists has been employed for segmentation tasks; however, inter-reader and intra-reader variability pose concerns regarding the consistency of those methods [10]. In such scenarios, test–retest repeatability offers a pseudo-standard for evaluating the reliability of features extracted from regions of interest (ROI) by assessing how consistently features are reproduced in sequential scans under similar conditions. Indeed, when there can be no histologic ground-state truth, repeatability may be the most important aspect of a segmentation algorithm to validate it for clinical use.

Prior studies have investigated the test–retest repeatability of quantitative features from PSMA-targeted ^68^Ga-PSMA and ^18^F-DCFPyL PET/CT datasets, utilizing semi-automated and/or manual segmentation [11,12]. Those studies have demonstrated, using statistical metrics, that while commonly used semi-quantitative parameters such as maximum standardized uptake value (SUV_max_) and mean standardized uptake value (SUV_mean_) exhibit relatively high repeatability, volumetric features such as PSMA-tumor volume and total lesion PSMA tend to show greater variability. Additionally, radiotracer uptake intensity has been correlated with repeatability, with higher uptake lesions demonstrating better, more robust repeatability [12]. However, when high-frequency radiomic features were analyzed, a substantial proportion exhibited poor repeatability, suggesting that feature robustness varies significantly depending on the type of features considered [13]. Given the increasing adoption of radiomics and artificial intelligence (AI)-derived features in oncologic imaging, establishing the reproducibility of such extracted features is crucial but unrealized.

With the advent of AI-driven methodologies in medical imaging, deep learning-based segmentation techniques have gained traction in automating lesion detection and delineation [14,15,16]. AI-guided segmentation offers several advantages, including reduction in observer bias, improved efficiency, and potential for enhanced reproducibility. Nonetheless, lesion-level test–retest repeatability of AI-assisted segmentation features has not been systematically analyzed in the context of PSMA-PET/CT imaging or PET imaging, more broadly. Understanding whether AI-assisted segmentation approaches can produce robust and repeatable quantitative and volumetric features is critical for their translation into routine clinical practice. In this context, the key contribution of the present study is a technical validation of AI-guided segmentation by assessing lesion-level test–retest repeatability of commonly used semi-quantitative and volumetric features on ^18^F-DCFPyL PSMA-PET/CT images. By focusing on repeatability under conditions where histologic ground truth is unavailable, this work provides a practical framework for evaluating the robustness of AI-guided segmentation outputs. The findings of this study have significant implications for clinical practice, as repeatable AI-extracted features could facilitate the development of reliable imaging biomarkers for disease monitoring, risk stratification, and treatment response assessment.

## 2. Materials and Methods

### 2.1. Study Design

**Figure 1:**
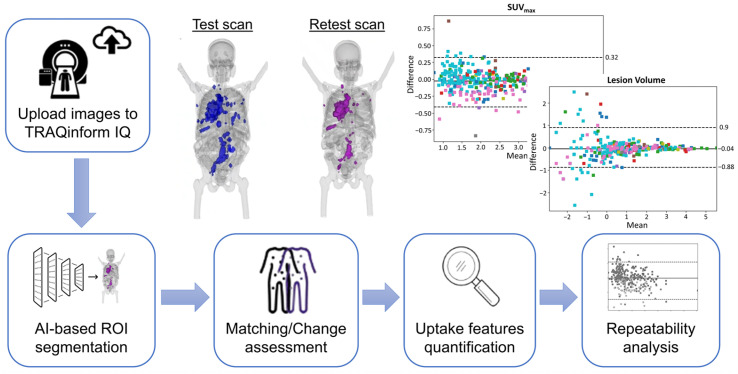
Workflow of the study from image upload to feature extraction and repeatability analysis.

This study utilized ^18^F-DCFPyL PSMA-PET/CT images in DICOM format from 22 patients diagnosed with metastatic PCa. All patients were originally accrued on an institutional review board-approved prospective protocol. PET/CT imaging was performed using a Siemens Biograph 128-slice mCT scanner (Siemens Healthineers, Erlangen, Germany) under standardized acquisition protocols to ensure reproducibility. Each patient underwent two PET/CT scans within a range of 1–7 days to assess test–retest repeatability, with no tumor-specific therapy administered between scans. Imaging was conducted approximately 60 min post-injection (range: 57–63 min) following intravenous administration of 322.4 MBq (first scan) and 323.6 MBq (second scan) of ^18^F-DCFPyL. PET data were acquired from mid-thigh to skull vertex over 6–8 bed positions (3 min per position), alongside a low-dose CT scan for attenuation correction and anatomical localization. Image reconstruction utilized the manufacturer’s ordered-subset expectation maximization algorithm, incorporating scatter and attenuation corrections. The study was registered at ClinicalTrials.gov (NCT03793543) and conducted under a United States Food and Drug Administration (FDA) Investigational New Drug Application (IND121064) as ^18^F-DCFPyL was not FDA-approved at the time. Detailed patient characteristics including age, prior treatments, and disease burden, as well as imaging acquisition protocols, have been previously published [12,17]. A graphical overview of the workflow for this study is shown in Figure 1.

### 2.2. AI-Guided Segmentation

**Figure 2:**
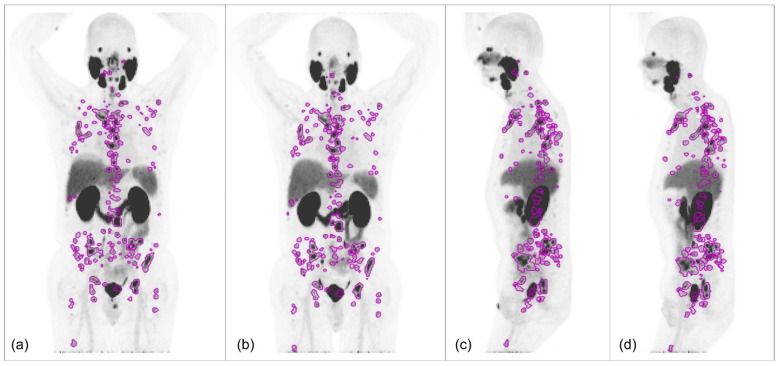
Test–retest ^18^F-DCFPyL PSMA-PET/CT maximum intensity projection images from a representative patient with metastatic prostate cancer. The images show AI-guided lesion segmentation (magenta outlines) on (**a**) test coronal, (**b**) retest coronal, (**c**) test sagittal, and (**d**) retest sagittal views. The retest scan was performed within 7 days of the initial scan.

Lesion segmentation from the images was performed using AI-guided TRAQinform IQ version 1.9 technology (AIQ Solutions, Madison, WI, USA). This software employed an automated lesion detection and segmentation algorithm based on a Retina U-Net architecture, applied to both baseline and follow-up scans [18]. Retina U-Net is a deep learning-based model that combines the RetinaNet object detection framework [19] with a U-Net segmentation architecture [20] to enhance lesion detection accuracy. Unlike conventional semantic segmentation approaches, Retina U-Net leverages a feature pyramid network [21] that enables multi-scale object detection while simultaneously integrating full-resolution segmentation supervision. That architecture refines object-level lesion detection by incorporating both pixel-wise and object-wise contextual information, improving accuracy in challenging medical imaging tasks [22]. The Retina U-Net was trained on a heterogeneous, multi-institutional patient dataset and evaluated using an external hold-out validation cohort that included both ^68^Ga-PSMA and ^18^F-labeled PSMA images. The software operated as a cloud-based service, where PET/CT images in DICOM format were uploaded to the platform and segmentation results were returned. The automated results were further refined through manual review by a quality assurance team led by a nuclear medicine technologist with over 22 years of experience to eliminate false-positive ROIs when these were located in healthy organs. A representative example of the AI-guided segmentation results, after expert refinement, is shown on test–retest maximum intensity projection images in Figure 2.

### 2.3. Feature Extraction

To track lesions over time, a registration-based method was implemented to match ROIs across the two time-points. That process involved first aligning the images from both scans and then determining which lesions corresponded between baseline and follow-up scans. This registration-based lesion matching approach has been previously validated and benchmarked against inter-reader variability, with no significant differences observed for precision, recall, F1-score, and the number of differences [23]. From the segmented and matched ROIs, the software extracted two semi-quantitative features (SUV_max_ and SUV_mean_) and two volumetric features (volume and SUV_total_) for each individual lesion at both time points. SUV_max_ represented the highest standardized uptake value (SUV) within the ROI, while SUV_mean_ indicated the average SUV within the ROI. Volume refers to the total size of the ROI measured in cm^3^, and SUV_total_ is determined by summing the SUVs of all voxels within the ROI and multiplying by the voxel volume.

### 2.4. Repeatability Analysis

To assess the test–retest repeatability of extracted features, multiple repeatability metrics including limits of agreement (LOA), intra-class correlation coefficients (ICC) [24] and within-subject coefficients of variations (wCOV) were calculated for all lesions. Additionally, Bland–Altman analysis was conducted to evaluate the limits of agreement of measurements and provide a visual representation of measurement differences and potential trends in variability [25]. Since SUV-based metrics often exhibit a skewed distribution, a natural log transformation was applied to the feature quantities to enhance statistical robustness before calculating the 95% limits of agreement (LOA) for the ratio between test and retest measurements. The final LOA values were then converted back using the formula:

$$95\%\;\mathrm{LOA}\;=\;\left(e^{\left(\bar{d}-1.96\sigma \right)},\;e^{\left(\bar{d}+1.96\sigma \right)}\right),$$

where bias $\bar{d}$ represented the mean ratio between test and retest measurements and σ was the standard deviation of the mean ratios [26]. Since many patients had multiple lesions with diverse radiotracer uptake levels, the calculation of $\bar{d}$ and σ followed the methodology designed for estimating limits of agreement in scenarios involving multiple observations per individual, where the true value may vary [27].

To assess the impact of lesion size on repeatability, additional analyses were performed by excluding ROIs below predefined volumetric thresholds. Specifically, repeatability metrics were recalculated, and Bland–Altman plots were recreated after excluding lesions smaller than 1 cm^3^ and 1.5 cm^3^, as smaller lesions are more susceptible to segmentation variability. These thresholds were selected based on prior PET imaging literature, where similar lesion size cut-offs have been used when evaluating feature repeatability and disease progression [26,28]. All statistical analyses to evaluate repeatability in this study were performed with Python version 3.9.

## 3. Results

### 3.1. Repeatability Across All Lesions

A total of 297 matching lesions of varying volumes were delineated using TRAQinform IQ software, with 191 lesions having a volume greater than 1 cm^3^ and 161 exceeding 1.5 cm^3^. To evaluate the test–retest repeatability of SUV_max_, SUV_mean_, SUV_total_, and volume, 95% limits of agreement, ICC and wCOV were computed. Table 1 presents these metrics across all lesions, as well as for lesions exceeding 1 cm^3^ and 1.5 cm^3^, respectively. The 95% LOA for each uptake parameter, as presented in Table 1 in percentage, indicates the expected range within which the percentage differences between test and retest measurements lie for 95% of the lesions. Those values account for inherent sources of variability in PET-based lesion quantification, including imaging noise, segmentation inconsistencies, and physiological fluctuations.

### 3.2. Effects of Lesion Size

The results demonstrate that lesion size plays a crucial role in test–retest agreement, with smaller lesions exhibiting greater variability. As shown in Table 1, excluding lesions smaller than 1 cm^3^ results in narrower LOA ranges and lower wCOV values, indicating improved measurement repeatability. This effect becomes more pronounced when considering only lesions greater than 1.5 cm^3^, where the LOA range further contracts and wCOV is further reduced in most of the features, indicating increased reliability of SUV and volume measurements in larger lesions.

### 3.3. Bland–Altman Analysis

**Figure 3:**
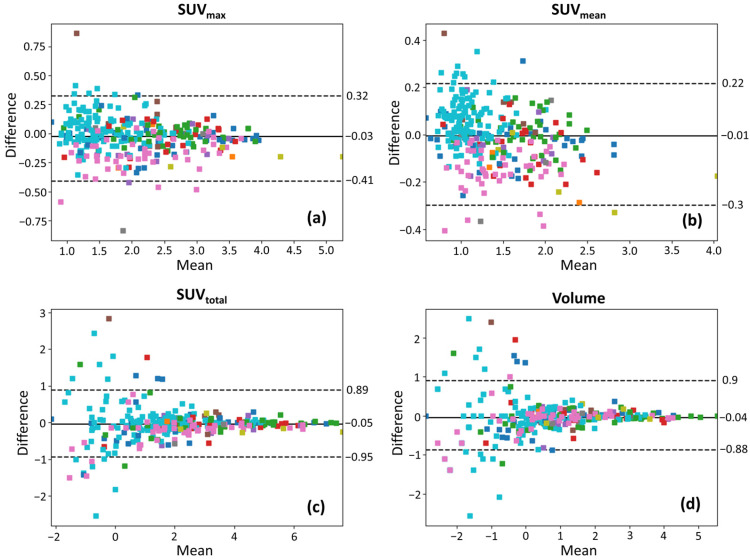
Bland–Altman plots on log-log scales, illustrating the test–retest variability of (**a**) SUV_max_, (**b**) SUV_mean_, (**c**) SUV_total_, and (**d**) lesion volume for all lesions. The solid line represents the mean difference, and dashed lines indicate the 95% limits of agreement. Data points are color-coded to represent different patient IDs.

**Figure 4:**
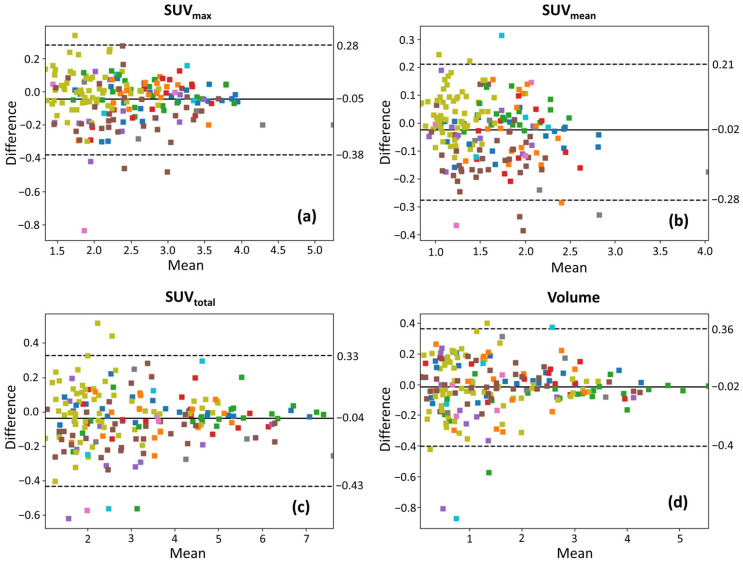
Bland–Altman plots on log-log scales, illustrating the test–retest variability of (**a**) SUV_max_, (**b**) SUV_mean_, (**c**) SUV_total_, and (**d**) lesion volume for lesions larger than 1 cm^3^. The solid line represents the mean difference, and dashed lines indicate the 95% limits of agreement. Data points are color-coded to represent different patient IDs.

**Figure 5:**
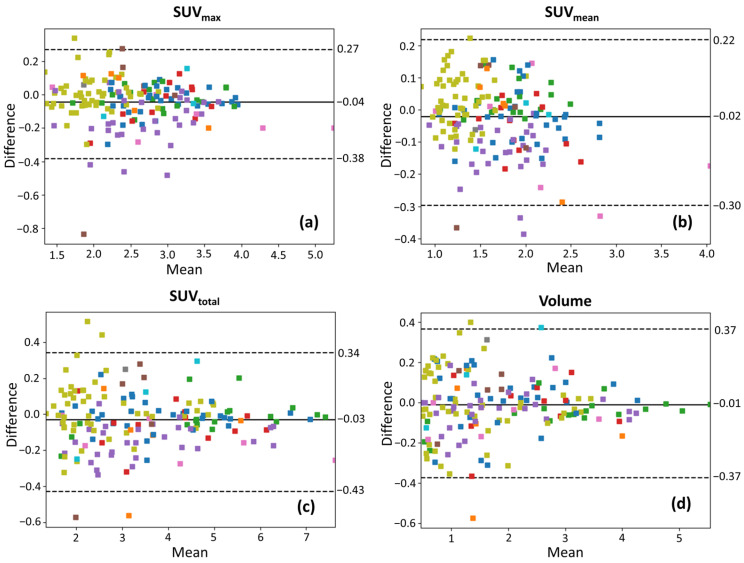
Bland–Altman plots on log-log scales, illustrating the test–retest variability of (**a**) SUV_max_, (**b**) SUV_mean_, (**c**) SUV_total_, and (**d**) lesion volume for lesions larger than 1.5 cm^3^. The solid line represents the mean difference, and dashed lines indicate the 95% limits of agreement. Data points are color-coded to represent different patient IDs.

Bland–Altman plots of the selected features are presented in Figure 3 on log-log scales for all lesion ROIs, in Figure 4 for ROIs with volume > 1 cm^3^, and in Figure 5 for ROIs with volume > 1.5 cm^3^, with color coding corresponding to individual patients. In those plots, the mean difference (bias) is close to zero across all uptake parameters, suggesting no significant systematic overestimation or underestimation between test and retest scans. The spread of the LOAs indicates variability in repeat measurements. Smaller lesions show a wider spread of differences (higher variability) than larger lesions, suggesting increased measurement uncertainty in smaller lesions. For SUV_max_ and SUV_mean_, the differences between test and retest scans remain relatively more stable across lesion sizes, though some heteroscedasticity is observed, with greater variability in smaller lesions. In contrast, SUV_total_ and volume exhibit more pronounced spread in smaller lesions, which suggests that volume-dependent effects influence test–retest variability for these lesions that have been segmented via AI assistance. In Figure 4 and Figure 5, the LOAs are narrower, further confirming that repeatability improves with larger lesion sizes.

## 4. Discussion

### 4.1. Semi-Quantitative vs. Volumetric Feature

This study demonstrates that SUV_max_, SUV_mean_, SUV_total_, and lesion volume exhibit test–retest variations of approximately ±30–40% (Table 1). This suggests that small changes in these metrics may fall within normal variability rather than reflecting true treatment response. ICC remains consistently high (>0.94) across all lesion groups and features in Table 1, suggesting that despite variability in individual lesion measurements, there is a strong overall agreement between test and retest scans. Compared to SUV_max_ and SUV_mean_, SUV_total_ and lesion volume exhibit higher wCOV and broader LOA ranges, which indicates that volumetric features are less repeatable than semi-quantitative SUV features.

Bland–Altman plots reveal distinct patient-dependent biases in SUV_max_ and SUV_mean_. For example, in Figure 3a,b, cyan-colored points skew positive while magenta-colored points skew negative with respect to the mean difference lines, suggesting systemic differences between patients. Potential explanations for these biases include variations in administered dose, tracer uptake kinetics, patient positioning, and physiological factors. However, patient-dependent biases appear to primarily influence SUV_max_ and SUV_mean_ but are less pronounced in SUV_total_ and volume, suggesting that volumetric parameters may be less influenced by these sources of variability. This implies that SUV_total_ variability may arise from two distinct sources: systemic PET measurement fluctuations (affecting SUV_mean_) and segmentation-derived volume differences. Future quantitative analysis will be needed to quantify the contributions of these two factors to SUV_total_ variability.

### 4.2. Lesion Size Dependence of Repeatability

Small lesions exhibit a unique pattern in volumetric measurement variability in Bland–Altman plots, evident in Figure 3c,d, where the variability increases abruptly, rather than gradually, as the lesion size decreases. This indicates a potential threshold effect, likely due to voxel quantization. Because small lesions contain a limited number of voxels, volume estimation becomes highly sensitive to even slight variations in segmentation. This effect introduces a “noise floor”, where minor changes in segmentation lead to disproportionately large differences in measured volume. Future work could involve reporting lesion sizes in both voxel count and physical units, as well as categorizing lesions into discrete voxel-based size bins, to provide a more precise evaluation of segmentation repeatability across different lesion sizes.

This study further shows that smaller lesions exhibit greater test–retest variability, making SUV-derived features from these lesions less stable. For widely metastatic patients undergoing systemic therapy and for whom response assessment is desired, machine learning models trained on PET imaging should consider excluding small lesions or prioritizing larger, more stable lesions, such as those exceeding 1.5 cm^3^, to enhance robustness. Additionally, feature selection techniques that account for variability should be incorporated to ensure that only highly repeatable features contribute to model predictions. For patients with low-volume metastatic disease, whose tumors may be small and/or subtle, existing approaches may need to be significantly refined to ensure repeatability and robustness, given the poor outcomes that can arise from failing to effectively treat all of the lesions in such patients [29].

### 4.3. Comparison with Manual Segmentation

Our AI-guided segmentation approach resulted in repeatability that was comparable to our previously reported manual segmentation-based analysis on the same test–retest cohort [12,17]. For example, the wCOV for SUV_max_ and SUV_mean_ in the current study, considering all the lesions, are 9.13% and 9.42%, similar to previously reported manual results (12.1% and 7.3%) [12]. While repeatability is comparable, the AI method offers clear advantages in processing efficiency and eliminates inter- and intra-reader variability.

### 4.4. Implications for Response Assessment

Quantitative PET imaging provides essential biomarkers for tumor detection, treatment response assessment, and prognostication [30]. However, inherent test–retest variability in PET-derived parameters must be carefully considered when defining thresholds for response classification. The observed negative percentage LOAs (Table 1) suggest that minor reductions in these values may reflect normal variability rather than actual disease regression. SUV_total_ and lesion volume show the highest test–retest variability in small lesions, which can affect prognostic models that rely on tumor burden quantification. To improve the reliability of volumetric PET biomarkers, excluding lesions below a threshold (e.g., 1.5 cm^3^) could enhance consistency. Standardizing PET-based biomarkers and refining response assessment frameworks like PERCIST or RECIP to integrate lesion size-specific adjustments will be critical for improving the reliability of PET imaging in both clinical and research settings [31,32]. In the context of metastatic disease, where a histologic ground truth is unlikely to ever be realistically available, lesion segmentation algorithms might best be judged by their repeatability. The more repeatable the algorithm’s semi-quantitative and volumetric outputs, the more robust the predictive and prognostic biomarkers derived from that algorithm should be.

Furthermore, the variability in the robustness of AI-assisted segmentation based on lesion size suggests that the current algorithm is best suited for delineating prominent disease sites in patients who are appropriate candidates for systemic therapy. However, patients with lower disease burden and more subtle metastatic lesions may require an algorithm optimized for detecting smaller lesions with lower SUV parameters. Interestingly, our findings align with those from a recent meta-analysis of ^18^F-FDG-PET studies [33], which demonstrated that lesions with higher uptake values showed better repeatability in terms of percent fluctuation across SUV metrics. This is consistent with our observations in PSMA-PET, where relative variability (as measured by wCOV) decreased with increasing SUV and lesion volume. These findings reinforce the broader principle that small or low-uptake lesions are more susceptible to biological and technical variability, regardless of the radiotracer used.

### 4.5. Limitations

The limitations of this study include that it is a post hoc analysis of a prospectively acquired dataset as opposed to a prospective and powered study to meet a defined endpoint. Nonetheless, this analysis was carried out with the largest PSMA-PET/CT test–retest dataset yet reported and obtaining larger datasets may be cost-prohibitive or impractical. Further, there are parameters yet to be explored such as whether there are metastasis-location-dependent variations in the repeatability of semi-quantitative or volumetric parameters. In addition, repeatability metrics were computed at the lesion level rather than the patient level, which may underrepresent patient-level biological and technical variability. Furthermore, while repeatability is crucial, it does not necessarily indicate that a feature is always sensitive to treatment-induced changes. Prior research has introduced the concept of “response-to-repeatability” [34], highlighting the need to assess whether highly repeatable PET features are also effective for monitoring treatment response. Future studies in PSMA-PET could explore this relationship to refine PET-based biomarkers for clinical decision-making.

## 5. Conclusions

This study emphasizes the critical need for standardization in PSMA-PET imaging protocols to minimize variability and improve measurement reproducibility, even when using AI-driven algorithms. The results highlight the significance of accounting for test–retest variability, particularly when using features derived from AI-driven lesion segmentation. While volumetric PSMA-PET metrics exhibit substantial fluctuations between repeat scans, even in the absence of biological changes, larger lesions tend to offer more stable and reproducible measurements. This reinforces the need for size-dependent response assessment strategies. Future research should focus on improving AI-driven segmentation and feature extraction models by integrating uncertainty quantification methods that can identify and mitigate the impact of unstable features. Such models could generate voxel-wise or lesion-level confidence maps, allowing clinicians to selectively trust high-confidence segmentations while flagging ambiguous or variable regions for manual review or exclusion from downstream analysis. The use of multimodal imaging, such as PET/MRI fusion, may further enhance the robustness of PET-based biomarkers by providing complementary anatomical and functional insights. Additionally, developing adaptive thresholding techniques that adjust for lesion size and inherent measurement variability could refine response assessment criteria, making PET imaging more reliable for precision oncology applications.

## Figures and Tables

**Table 1:** Repeatability metrics of lesion uptake features for (A) all lesions, (B) lesions larger than 1 cm^3^ and (C) lesions larger than 1.5 cm^3^.

(A) All lesions (number of lesions: 297)				
	**SUV_max_**	**SUV_mean_**	**SUV_total_**	**Lesion Volume**
Lower LOA (%)	−33.81	−25.78	−61.34	−58.62
Upper LOA (%)	38.02	24.10	142.36	145.89
ICC	0.973	0.960	0.972	0.996
wCOV	9.13	9.42	22.22	63.67
(B) Lesions having volume > 1 cm^3^ (number of lesions: 191)				
Lower LOA (%)	−31.72	−24.27	−35.15	−33.07
Upper LOA (%)	32.31	23.38	38.48	43.83
ICC	0.972	0.958	0.974	0.996
wCOV	6.88	7.82	6.08	12.27
(C) Lesions having volume > 1.5 cm^3^ (number of lesions: 161)				
Lower LOA (%)	−31.82	−25.74	−34.88	−31.13
Upper LOA (%)	31.01	24.26	40.54	44.31
ICC	0.971	0.949	0.972	0.995
wCOV	6.50	7.90	5.62	10.34

## Data Availability

Data are available to serious investigators upon reasonable request.

## Abbreviations

The following abbreviations are used in this manuscript:

PET	positron emission tomography
PSMA	prostate-specific membrane antigen
SUV	standardized uptake value
LOA	limits of agreement
ROI	region of interest
